# Supplementary material for: Investigating the Joint Effect of Allostatic Load among Lesbian, Gay, and Bisexual Adults with Risk of Cancer Mortality
Source: Int J Environ Res Public Health. 2023 Jun 13;20(12):6120. doi: 10.3390/ijerph20126120 (PMC10298095; doi:10.3390/ijerph20126120)
Supplement: Supplementary file 1 [file ijerph-20-06120-s001.zip › ijerph-2280616-supplementary.pdf]

Table S1: Weighted distribution of allostatic load components by sex reported at survey, NHANES years 2001 through 2010, among 12,470 participants. Thresholds for each allostatic load component based on these weighted distributions.

**Allostatic Load Component**	**Median (Q1, Q3) [a]**
**Albumin, serum (g/dL)**	
Female	4.15 (3.95, 4.35)
Male	4.37 (4.18, 4.55)
**Body Mass Index (kg/m^2)**	
Female	26.63 (22.81, 32.24)
Male	27.58 (24.50, 31.11)
**C-reactive Protein (mg/dL)**	
Female	0.20 (0.06, 0.51)
Male	0.13 (0.06, 0.30)
**Creatinine (μmol/L)**	
Female	65.04 (61.03, 71.65)
Male	87.05 (78.69, 96.37)
**Glycohemoglobin (%)**	
Female	5.21 (5.00, 5.47)
Male	5.28 (5.05, 5.52)
**Systolic Blood Pressure (mmHg)**	
Female	112.92 (104.60, 123.92)
Male	119.44 (111.94, 128.29)
**Diastolic Blood Pressure (mmHg)**	
Female	69.43 (62.32, 76.54)
Male	72.69 (65.15, 80.25)
**Total cholesterol (mg/dL)**	
Female	192.38 (167.93, 220.16)
Male	195.67 (169.52, 222.90)
**Triglycerides, serum (mg/dL)**	
Female	98.09 (67.20, 145.21)
Male	126.83 (83.28, 204.38)

[a] Estimated using sampling weights from National Health and Nutrition Examination Survey (NHANES).
Q1 = 25th percentile, Q3 = 75th percentile.

Table S2: Survey weighted Cox proportional hazard models presented as Hazard Ratios (HR) and 95% Confidence Intervals (CI) for the association between sexual orientation/allostatic load and risk of cancer death, among 12,470 (weighted *N* = 122,729,451) NHANES participants with 222 (weighted n = 1,955,041) cancer-related deaths.

	No. & (Weighted %) Cancer Deaths	Mean Survival Months (SE)	Hazard Ratio (HR) and 95% Confidence Interval (CI)	
			Model 1	Model 2
**Sexual Orientation and Allostatic Load Status**				
Straight/heterosexual with low allostatic load	78 (1.1)	207.6 (0.2)	1.00 (Referent)	1.00 (Referent)
Straight/heterosexual with high allostatic load	128 (2.3)	218.3 (0.3)	1.22 (0.81 – 1.84)	1.06 (0.68 – 1.66)
Gay/lesbian with low allostatic load	1 (0.6)	Unreliable	0.60 (0.08 – 4.76)	0.66 (0.08 – 5.23)
Gay/lesbian with high allostatic load	4 (7.5)	154.8 (1.5)	4.94 (1.99 – 12.27)	4.17 (1.78 – 9.79)
Bisexual with low allostatic load	2 (0.6)	110.5 (0.8)	0.99 (0.24 – 4.11)	0.70 (0.17 – 2.97)
Bisexual with high allostatic load	2 (2.7)	110.9 (0.1)	2.47 (0.58 – 10.55)	2.30 (0.52 – 10.11)
MSM & WSW with low allostatic load	2 (0.5)	118.5 (0.7)	0.48 (0.11 – 2.06)	0.44 (0.10 – 1.88)
MSM & WSW with high allostatic load	5 (4.9)	162.1 (1.7)	3.05 (1.62 – 5.74)	2.07 (1.09 – 3.90)
**Among those living with high allostatic load**				
Straight/heterosexual	128 (2.3)	218.3 (0.3)	1.00 (Referent)	1.00 (Referent)
Gay/lesbian	4 (7.5)	154.8 (1.5)	3.90 (1.62 – 9.41)	3.61 (1.66 – 7.88)
Bisexual	2 (2.7)	110.9 (0.1)	1.95 (0.48 – 7.91)	2.03 (0.49 – 8.40)
MSM & WSW	5 (4.9)	162.1 (1.7)	2.48 (1.28 – 4.80)	1.85 (0.97 – 3.52)
**Among those living with low allostatic load**				
Straight/heterosexual	78 (1.1)	207.6 (0.2)	1.00 (Referent)	1.00 (Referent)
Gay/lesbian	1 (0.6)	Unreliable	0.61 (0.08 – 4.91)	0.73 (0.10 – 5.60)
Bisexual	2 (0.6)	110.5 (0.8)	1.05 (0.25 – 4.42)	0.73 (0.17 – 3.17)
MSM & WSW	2 (0.5)	118.5 (0.7)	0.47 (0.11 – 2.04)	0.43 (0.10 – 1.88)
p-value for interaction between sexual orientation and allostatic load			0.06	0.06

Percentages are weighted. Cox proportional hazard models are estimated using NHANES survey weighting.
(MSM) men who have sex with men & (WSW) women who have sex with women are participants in the absence of a current lesbian, gay or bisexual identity.
Model 1 is adjusted for age.
Model 2 is adjusted for age, and sociodemographic factors including reported sex at survey, race, income, education, smoking status, and alcohol consumption.

Table S3: **Fine & Gray method for proportional hazard models presented as Sub-Distribution Hazard ratios (SHR) and 95% Confidence Intervals (CI) for the association between allostatic load and risk of cancer death, among 12,470 (weighted *N* = 122,729,451) NHANES participants with 222 (weighted n = 1,955,041) cancer-related deaths, accounting for 484 (weighted n = 4,472,101) competing all-cause deaths.**

	**No. & (Weighted %) Cancer Deaths**	**No. & (Weighted %) All-Cause Deaths**	**Mean Survival Months (SE)**	**Sub-Distribution Hazard Ratio (SHR) and 95% Confidence Interval (CI)**	
				Model 1	Model 2
**Sexual Orientation and Allostatic Load Status**					
Straight/heterosexual with low allostatic load	78 (1.1)	169 (2.4)	207.6 (0.2)	1.00 (Referent)	1.00 (Referent)
Straight/heterosexual with high allostatic load	128 (2.3)	280 (5.5)	218.3 (0.3)	1.20 (0.89 – 1.61)	1.08 (0.80 – 1.47)
Gay/lesbian with low allostatic load	1 (0.6)	4 (3.7)	Unreliable	0.74 (0.10 – 5.41)	0.80 (0.11 – 5.79)
Gay/lesbian with high allostatic load	4 (7.5)	3 (3.9)	154.8 (1.5)	3.74 (1.39 – 10.07)	3.03 (1.11 – 8.26)
Bisexual with low allostatic load	2 (0.6)	4 (1.7)	110.5 (0.8)	1.49 (0.36 – 6.13)	1.12 (0.28 – 4.51)
Bisexual with high allostatic load	2 (2.7)	14 (10.7)	110.9 (0.1)	1.20 (0.29 – 4.94)	1.07 (0.26 – 4.44)
MSM & WSW with low allostatic load	2 (0.5)	2 (0.5)	118.5 (0.7)	0.89 (0.22 – 3.68)	0.86 (0.21 – 3.55)
MSM & WSSW with high allostatic load	5 (4.9)	8 (4.1)	162.1 (1.7)	1.61 (0.64 – 4.03)	1.29 (0.50 – 3.28)
p-value for interaction between sexual orientation and allostatic load				0.57	0.71

Percentages are weighted. Cox proportional hazard models are estimated using NHANES survey weighting.
(MSM) men who have sex with men & (WSW) women who have sex with women are participants in the absence of a current lesbian, gay or bisexual identity.
Model 1 is adjusted for age.
Model 2 is adjusted for age, and sociodemographic factors including reported sex at survey, race, income, education, smoking status, and alcohol consumption.

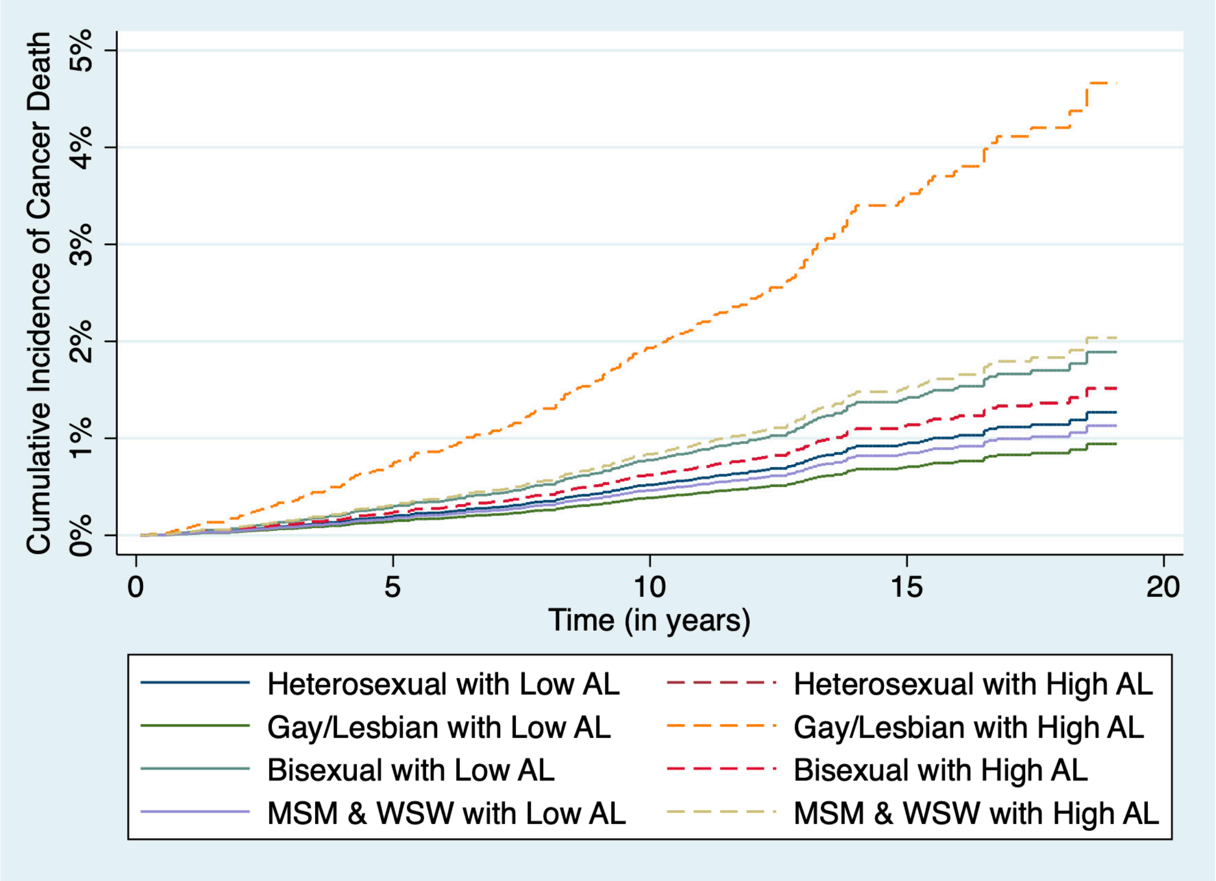

Figure S1: Unweighted age adjusted cumulative incidence function competing risks plots for time to cancer death by sexual orientation and allostatic load status. (MSM) men who have sex with men & (WSW) women who have sex with women are participants in the absence of a current lesbian, gay or bisexual identity.
